# B cell OX40L supports T follicular helper cell development and contributes to SLE pathogenesis

**DOI:** 10.1136/annrheumdis-2017-211499

**Published:** 2017-08-17

**Authors:** Andrea Cortini, Ursula Ellinghaus, Talat H Malik, Deborah S Cunninghame Graham, Marina Botto, Timothy James Vyse

**Affiliations:** 1 Division of Medical and Molecular Genetics and Immunology, Infection and Inflammatory Disease, King’s College London, London, UK; 2 Department of Medicine, Centre for Complement and Inflammation Research, Imperial College London, London, UK

**Keywords:** systemic lupus erythematosus, B cells, OX40L, T follicular helper cells, autoantibodies

## Abstract

**Objectives:**

*TNFSF4* (encodes OX40L) is a susceptibility locus for systemic lupus erythematosus (SLE). Risk alleles increase *TNFSF4* expression in cell lines, but the mechanism linking this effect to disease is unclear, and the OX40L-expressing cell types mediating the risk are not clearly established. Blockade of OX40L has been demonstrated to reduce disease severity in several models of autoimmunity, but not in SLE. We sought to investigate its potential therapeutic role in lupus.

**Methods:**

We used a conditional knockout mouse system to investigate the function of OX40L on B and T lymphocytes in systemic autoimmunity.

**Results:**

Physiologically, OX40L on both B and T cells contributed to the humoral immune response, but B cell OX40L supported the secondary humoral response and antibody affinity maturation. Our data also indicated that loss of B cell OX40L impeded the generation of splenic T follicular helper cells. We further show that in two models of SLE—a spontaneous congenic model and the H2-IA^bm12^ graft-versus-host-induced model—loss of B cell OX40L ameliorates the autoimmune phenotype. This improvement was, in each case, accompanied by a decline in T follicular helper cell numbers. Importantly, the germline knockout did not exhibit a markedly different phenotype from the B cell knockout in these models.

**Conclusions:**

These findings contribute to a model in which genetically determined increased OX40L expression promotes human SLE by several mechanisms, contingent on its cellular expression. The improvement in pathology in two models of systemic autoimmunity indicates that OX40L is an excellent therapeutic target in SLE.

## Introduction

Systemic lupus erythematosus (SLE) is a chronic autoimmune disease characterised by autoantibodies against nuclear antigens along with the deposition of immune complexes.^1 2^ As with most other autoimmune diseases, environmental and genetics factors contribute to the risk of developing SLE. Genome-wide association studies have revealed over 50 susceptibility loci.^3 4^ *TNFSF4* (tumour necrosis factor ligand family, member 4, CD252) is an established susceptibility gene for SLE^4 5^ and for several other autoimmune diseases.^6–9^ Fine-mapping of this locus in SLE identified two independent association signals upstream of *TNFSF4* in multiple ancestries.^10^ These two signals align with separate expression quantitative trait loci, each one associated with elevated expression of *TNFSF4* in Epstein Barr virus (EBV) lymphoblastoid cell lines,^11^ suggesting that *TNFSF4* transcription is upregulated in individuals harbouring risk alleles.

*TNFSF4* encodes the costimulatory molecule, OX40L, a type II transmembrane protein expressed on several immune cell types on activation, including anitigen presenting cells (APCs), such as dendritic cells (DCs), B cells and macrophages,^12–14^ activated T cells,^15 16^ and mast cells and vascular endothelial cells.^17^ In contrast, its only known receptor, OX40, is expressed mainly on activated CD4+ T cells.^18–21^ The OX40L-OX40 signalling pathway is fundamental for effector T cell proliferation and memory T cell development, maintenance of cytokine production by T cells and DCs, increasing Ig production, and promoting plasma cell development.^15 22–27^ Nevertheless, how these various functions relate to the cell types expressing OX40L is still unclear. Constitutive expression of OX40L on T cells has been shown to induce spontaneous autoimmunity in C57BL/6 mice.^23^ A recent study showed that OX40L expression on a subset of myeloid DCs is implicated in the pathogenesis of SLE.^28^ The beneficial effect of blocking the OX40L-OX40 signalling pathway has been shown in several different mouse models of autoimmune diseases,^17^ but experimental evidence of its efficacy in SLE is unknown.

We sought to understand the function of OX40L using CD4+ T cell and B cell conditional knockout mice. We investigated the role of OX40L using immunisation and we went on to determine how the loss of OX40L affected the pathology in two different SLE mouse models.

## Materials and methods

### Mice

A bacterial artificial chromosome (BAC) clone encoding the extracellular domain and 3′-untranslated region of *Tnfsf4* was obtained from a C57BL/6-derived genomic library. The *Tnfsf4* conditional targeting vector was constructed using recombineering,^29^ as described in online supplementary figure S1A. The mice (*Tnfsf4*^fl/fl^) were made according to a standard gene targeting approach in A9 embryonic stem cells (ES). We used (129xC57BL/6)F1 ES; therefore, microsatellite analyses were undertaken to confirm that the targeting vector had recombined on the C57BL/6 chromosome. The mice were backcrossed for eight generations on the C57BL/6 background. *Tnfsf4*^fl/fl^ mice were crossed with β-actin-cre, CD4-cre and CD19-cre (Jackson Laboratories) to obtain *Tnfsf4*^−/−^, *Tnfsf4*^fl/fl^/CD4-cre and *Tnfsf4*^fl/fl^/CD19-cre, respectively. Before each experiment mice were genotyped by PCR. The primers and expected PCR product size are listed in supplementary table S1. B6.*Sle16* mice were bred in-house and B6.*Sle16.Tnfsf4*^−/−^ were generated by crossing them with *Tnfsf4*^−/−^ mice. B6-H2^bm12^ mice were purchased from the Jackson Laboratories (B6(C).*H2-Ab1^bm12^*/KhEgJ; strain no. 001162, https://www.jax.org/strain/001162). The mice used were female, 8–12 weeks old and housed in specific pathogen-free conditions. All animal procedures were performed in accordance with institutional guidelines and approved by the UK Home Office.

10.1136/annrheumdis-2017-211499.supp1 Supplementary file 1

### In vitro analysis of OX40L expression

To assess OX40L expression in vitro, different cell subsets were purified from mouse spleen using LS Columns and MACS Technology (Miltenyi Biotec) and stimulated as described before.^16 24^ Briefly, single cell suspensions were obtained from collagenase-treated spleens, and B cells, DCs and T cells were then purified incubating the splenic cell suspension with anti-CD43(Ly-48) microbeads, anti-CD11c microbeads or CD4+ T cell isolation kit, respectively, following the manufacturers’ protocols. The purity of each subpopulation was tested routinely by fluorescence-activated cell sorting (FACS) and a value >95% was measured for each purification. Purified B cells and DCs were stimulated for 72 hours with anti-CD40 (Clone3/23 at 2.5 µg/mL) plus F(ab’)2 anti-mouse IgM (10 µg/mL) or anti-CD40 alone, respectively. T cells were stimulated with anti-CD3 (0.005 µg/mL), IL-2 (100 U/mL) and IL-12 (10 ng/mL) for 7 days. After stimulation, the cells were harvested and analysed by FACS for OX40L expression.

### Flow cytometry

Flow cytometry was performed using a five-colour or six-colour staining protocol and analysed with a BD FACSVerse (BD Biosciences, San Jose, California, USA). The following Abs were used: anti-CD4 (GK.5), anti-CXCR5 (L138D7), anti-PD1(29F.1a12), anti-CD62L (MEL-14), anti-CD44 (IM7), anti-B220 (RA3-6B2), anti-GL7 (GL7), anti-CD138 (281-2) and anti-IgD (11–26 c.2a). Abs were purchased from BioLegend (San Diego, California, USA). Staining was performed in the presence of a saturating concentration of 2.4G2 mAb (anti-FcγRII/III). Data were analysed using FlowJo V.9 (Tree Star, Ashland, Oregon, USA).

### Immunisation and ELISA

Mice were immunised subcutaneously with 50 μg 4-hydroxy-3-nitrophenylacetyl-chicken gamma globulin (NP-CGG) in complete Freund’s adjuvant. For the analysis of the secondary response mice were reimmunised with 50 µg of NP-CGG in incomplete Freund’s adjuvant 35 days after receiving the first immunisation. Serum was collected on days 7, 14, 28 and 42, and titres of isotype-specific low-affinity and high-affinity antibodies to NP were measured by ELISA in plates coated with either NP25-BSA or NP4-BSA (4-hydroxy-3-nitrophenylacetyl hapten conjugated to bovine serum albumin), respectively.^30^ Briefly NUNC plates were coated with the antigen at 5 µg/mL in borate buffered saline (BBS) overnight at 4°C. Plates were washed with phosphate buffered saline (PBS) and then blocked for 1 hour at room temperature with 0.5% BSA in PBS. Samples were diluted in dilution buffer (PBS 2%,  bovine serum albumin (BSA) 0.05% Tween-20) and added, in duplicate, to the plates for 3 hours at 37°C. Plates were washed and incubated with alkaline phosphatase (AP)-conjugated secondary antibody specific for the different Ig isotype (SouthernBiotech) for 3 hours at room temperature. Plates were developed with p-nitrophenylphosphate (Sigma). A standard serum was generated from a pool of reactive serum of immunised wild-type mice. Absorbance was read at 405 nm and data were expressed as arbitrary ELISA unit (AEU) in reference to a standard curve obtained by serial dilution of the standard serum.

### cGvHD mouse model and autoantibody assays

Knockout and control mice were injected intraperitoneally with 5×10^7^ splenocytes from B6.*H2^bm12^* mice. Briefly, splenocytes were obtained as a single cell suspension by mashing the spleen collected through 70 µm cell strainers using the plunger from a syringe. After lysis of the red blood cells, splenocytes were counted and resuspended at 5×10^8^ cells/mL in PBS and 100 µL was injected in each mouse. Serum was collected on days 14, 28 and 42, and titres of IgG antibodies to double-stranded deoxyribonucleic acid (dsDNA) were measured by ELISA using dsDNA (100 µg/mL) or single-stranded deoxyribonucleic acid (ssDNA) (10 µg/mL) in BBS buffer as coating antigen. Bound Abs were detected with AP-conjugated goat anti-mouse IgG (-chain specific) (Sigma-Aldrich) or IgM (Southern Biotechnology Associates). The results were expressed as AEU relative to a standard positive sample derived from an MRL/Mp*^lpr/ lpr^* mice pool.

### Total serum IgG and IgM levels

Total serum IgM and IgG levels were assayed by capture ELISA as previously described.^31^

### IgG, IgM and C3 kidney deposition

Fluorescein (FITC)-conjugated goat Abs against mouse total IgG (1/400 dilution; Sigma-Aldrich), mouse total IgM (1/200 dilution, eBioscience) and against mouse C3 (1/50 dilution; ICN Pharmaceuticals) were used on snap-frozen kidney sections. The staining with FITC-conjugated Abs was quantified as previously described^31^ and expressed as arbitrary fluorescence units.

### Statistical analysis

Where appropriate either the Student’s t-test, two-way analysis of variance (ANOVA) or one-way ANOVA followed by Fisher’s least significant difference (LSD) multiple comparison test was performed using GraphPad Prism V.6.00 for Windows (GraphPad Software, La Jolla, California, USA).

## Results

### Generation of *Tnfsf4* conditional knockout strains

We generated a floxed *Tnfsf4* mouse (*Tnfsf4^fl/fl^*) on the C57BL/6 genetic background (see online supplementary figure S1A,B) to avoid the confounding effects caused by epistatic interactions between 129 and C57BL/6 genes that promote an autoimmune phenotype.^32^ Germline knockout (KO) mice were obtained by crossing *Tnfsf4^fl/fl^* with the β-actin *Actb-cre* mouse strain. Conditional T cell *Tnfsf4^fl/fl^(CD4)^−/−^* and B cell *Tnfsf4^fl/fl^(CD19)^−/−^* specific knockout mice were created by crossing with CD4-*cre*^33^ and CD19-*cre* mice,^34^ respectively. Lack of OX40L was observed in all cell types from *Tnfsf4^−/−^* mice, while a cell-specific deletion was confirmed in the conditional knockouts (see online supplementary figure S1C).

### B cell OX40L promotes antibody affinity maturation

**Figure 1:**
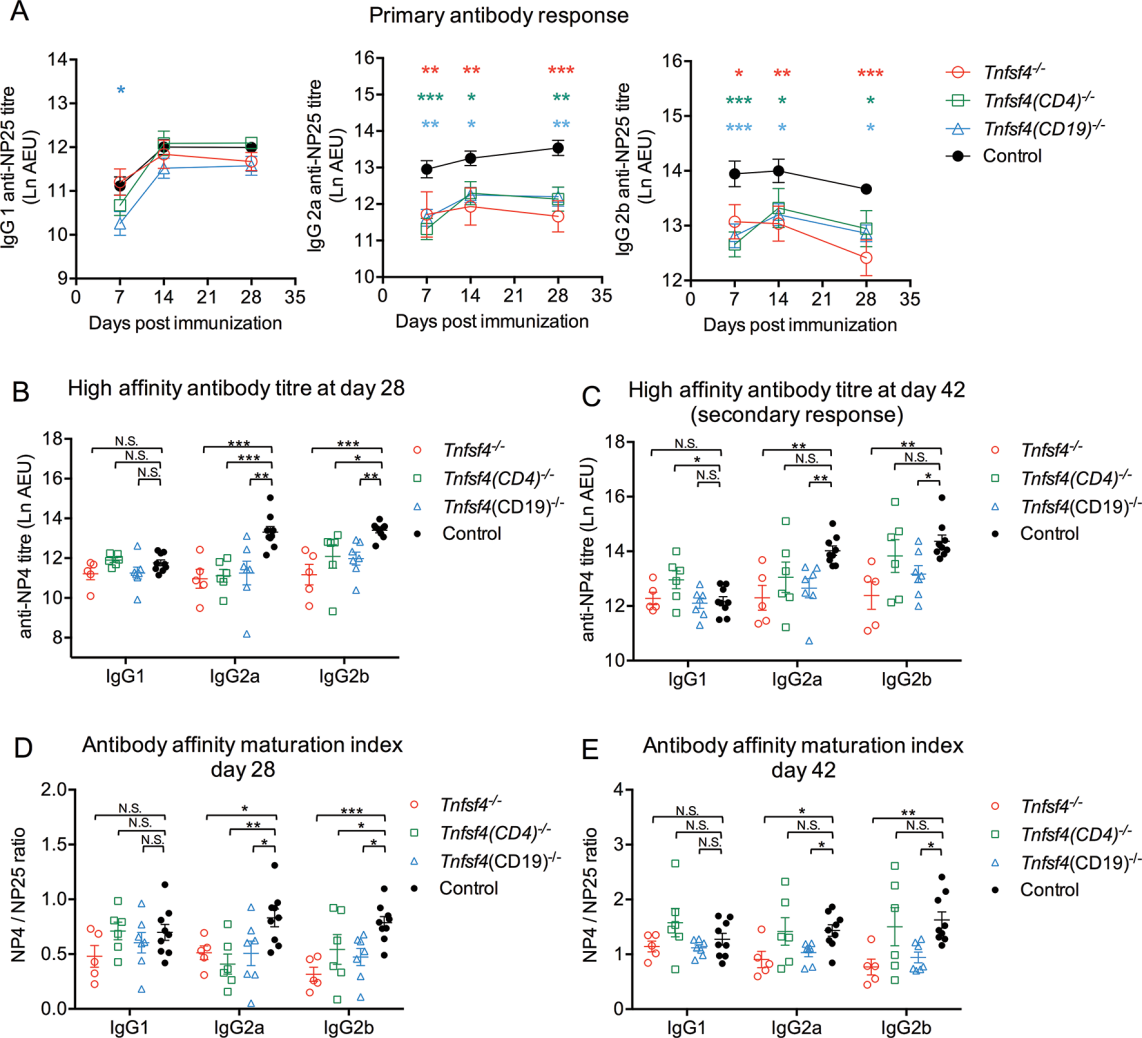
OX40L in T cell-dependent humoral response. Wild-type controls, *Tnfsf4*^−/−^, *Tnfsf4*^fl/fl^(CD19)^−/−^ and *Tnfsf4*^fl/fl^(CD4)^−/−^ mice were immunised with NP-CGG in CFA and reimmunised on day 35 with NP-CGG in IFA. Sera were collected on days 7, 14 and 28 for the primary response and on day 42 for the secondary response. (A) Titres of NP-specific low-affinity antibody measured with NP25-BSA. (B) Titres of NP-specific high-affinity antibodies on day 28 measured with NP4-BSA. (C) Titres of NP-specific high-affinity antibodies on day 42 measured with NP4-BSA. (D) Affinity maturation index calculated as ratio of the titres of IgG detected with NP4-BSA to those with NP25-BSA on day 28. (E) Affinity maturation index calculated as the ratio of the titres of IgG detected with NP4-BSA to those with NP25-BSA on day 42. Each symbol represents an individual mouse; dots in (A) and bars in (B–E) indicate the mean titre, each being shown mean±SEM. AEU is arbitrary ELISA unit; N.S. is not significant; *p<0.05, **p<0.01 and ***p<0.001 (A, two-way ANOVA; B–E, one-way ANOVA). ANOVA, analysis of variance; CFA, complete Freund’s adjuvant; IFA, incomplete Freund’s adjuvant; NP-CGG, nitrophenylacetyl-chicken gamma globulin; NP25-BSA, NP4-BSA, 4-Hydroxy-3-nitrophenylacetyl hapten conjugated to bovine serum albumin.

Conflicting data have been reported on the importance of the OX40L-OX40 pathway in controlling T-dependent antibody responses.^24 26 35^ Thus, we explored this response by immunising the three KO strains and a control group with NP-CGG, a well-studied T cell-dependent antigen. All three strains showed significantly lower titres of low-affinity IgG2a and IgG2b antibodies against NP25-BSA compared with wild-type mice (figure 1A). In contrast, the IgG1 response was hardly affected by the lack of OX40L. Affinity maturation during the primary response was also assessed by measuring antibody against NP4-BSA on day 28 and by calculating the affinity maturation index (ratio of high-affinity to low-affinity antibody responses). All three knockout strains displayed lower titres of high-affinity IgG2a and IgG2b (figure 1B,C) compared with wild-type animals and a lower affinity maturation index (figure 1D), which suggested OX40L contribution in the antibody affinity maturation process. To investigate the role of OX40L in the secondary immune response, mice were then boosted with NP-CGG on day 35, and the high-affinity antibody response was measured 1 week later. *Tnfsf4^−/−^* and *Tnfsf4^fl/fl^(CD19)^−/−^* mice both showed a significantly impaired IgG2a and IgG2b memory response compared with control mice, associated with a lower affinity maturation index (figure 1C,E). In contrast, the memory response in the *Tnfsf4^fl/fl^(CD4)^−/−^* mice was normal (figure 1C). These results indicate a role for both B and T cell OX40L in the primary immune response, with a distinct role for B cell OX40L in the affinity maturation of the secondary humoral immune response.

### OX40L is essential for T cell activation

**Figure 2:**
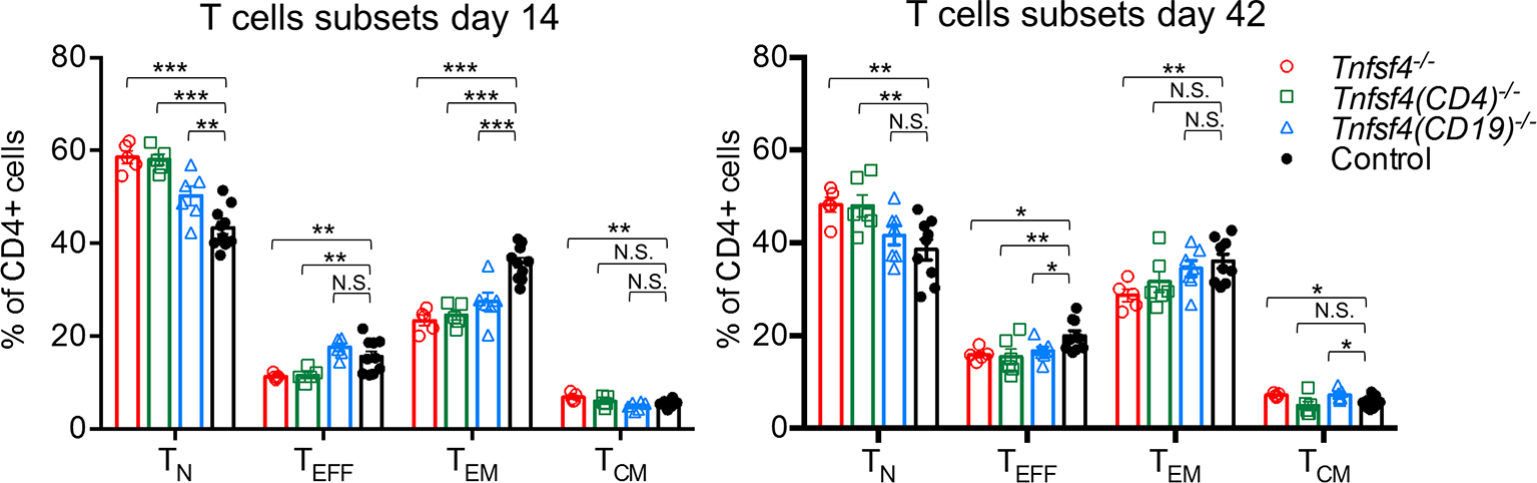
Role of OX40L in T cell activation. Wild-type controls, *Tnfsf4*^−/−^, *Tnfsf4*(CD19)^−/−^ and *Tnfsf4*(CD4)^−/−^ mice were immunised with NP-CGG in CFA and reimmunised on day 35 with NP-CGG in IFA. Spleens were taken and analysed by FACS either on day 14 or day 42. Quantification of naïve (CD4+, CD62L+, CD44^low^) T cells, effector (CD4+, CD62L^low^, CD44^low^) T cells, effector/memory (CD4+, CD62L^low/neg^, CD44^hi^) T cells and central/memory (CD4+, CD62L+, CD44^hi^) T cells on day 14 (left) and day 42 (right). Each symbol represents an individual mouse. Bars indicate the mean±SEM. N.S., not significant; *p<0.05, **p<0.01 and ***p<0.001 (one-way analysis of variance). CFA, complete Freund’s adjuvant; FACS, fluorescence-activated cell sorting; IFA, incomplete Freund’s adjuvant; NP-CGG, nitrophenylacetyl-chicken gamma globulin.

As the impaired humoral response could be a consequence of defective T cell activation, we decided to investigate the splenic T cell composition (see online supplementary figure S2A) of immunised mice on days 14 and 42 (figure 2). By day 14, *Tnfsf4^−/−^* and *Tnfsf4^fl/fl^(CD4)^−/−^* had a markedly lower proportion of effector T and effector/memory CD4+ T cells. In contrast, *Tnfsf4^fl/fl^(CD19)^−/−^* mice showed only a reduction in the proportion of effector/memory T cells, indicating that B cell OX40L may not play a major role in priming naïve T cells. On day 42, all three knockout strains had fewer T effector cells than control mice (figure 2). Interestingly, *Tnfsf4^−/−^* and *Tnfsf4^fl/fl^(CD19)^−/−^* mice also showed a small, but statistically significant, increment in the frequency of central memory T cells, suggesting that OX40L may regulate the balance between effector and central memory T cells during the recall response (figure 2). Our data confirmed the previous reported role of OX40-OX40L signalling in T cell activation and development of T effector memory cells.^18 22 23 36^ The explanation for the difference in the secondary humoral response between *Tnfsf4^fl/fl^(CD4)^−/−^* and *Tnfsf4^fl/fl^(CD19)^−/−^* mice was not evident. We therefore decide to investigate further analysing the extent of the germinal centre (GC) reaction in the immunised mice.

### OX40L on B cells supports plasma cell development

**Figure 3:**
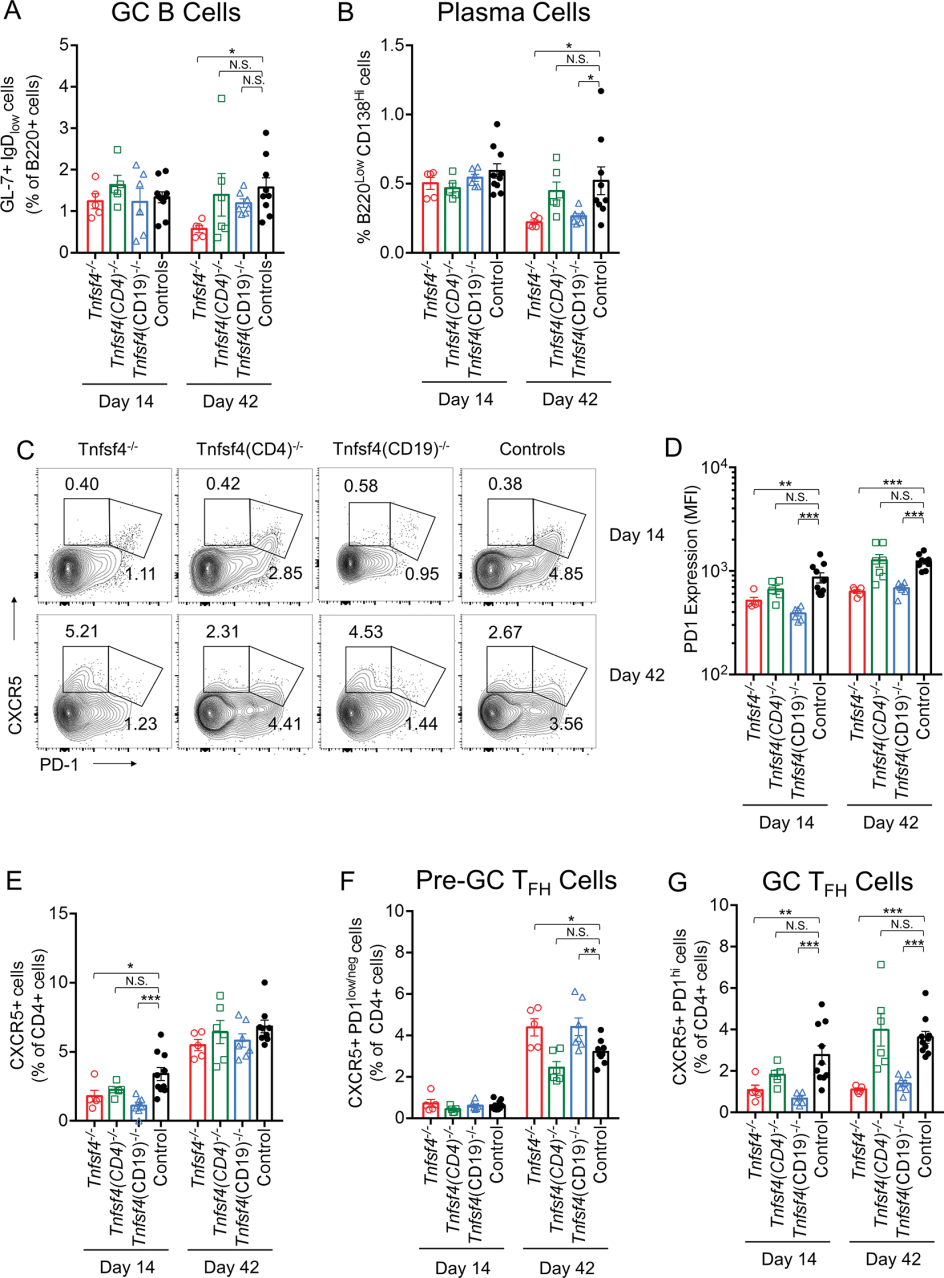
OX40L function in GC reaction. Wild-type controls, *Tnfsf4*^−/−^, *Tnfsf4*(CD19)^−/−^ and *Tnfsf4*(CD4)^−/−^ mice were immunised with NP-CGG in CFA and reimmunised on day 35 with NP-CGG in IFA. Spleens were taken and analysed by FACS either on day 14 or day 42. (A) Frequency of GC B cell (B220+, GL7+, IgD−) presented as frequency among the B220+ population. (B) Percentage of plasma cells (B220^low^, CD138^hi^). (C) Gating of T follicular helper (T_FH_) (CD4+, CXCR5+, PD-1^hi^) and pre-T follicular helper (pre-T_FH_) (CD4+, CXCR5+, PD-1^Low/neg^) cells. (D) PD-1 expression level in CD4+ cells assessed by FACS. (E) Frequency of CXCR5+ cells presented as frequency among the CD4+ population. (F) Quantification of pre-GC T_FH_ and (G) GC-T_FH_ cells as gated in (C) presented as frequency among the CD4+ population. Each symbol represents an individual mouse. Bars indicate the mean±SEM, N.S., not significant; *p<0.05, **p<0.01 and ***p<0.001 (one-way analysis of variance). CFA, complete Freund’s adjuvant; FACS, fluorescence-activated cell sorting; GC, germinal centre; IFA, incomplete Freund’s adjuvant; NP-CGG, nitrophenylacetyl-chicken gamma globulin.

All three groups of immunised KO mice showed no difference in the GC B cell population (see online supplementary figure S2B) on day 14 (figure 3A), although during the secondary response, 1 week after the rechallenge, *Tnfsf4^−/−^* mice showed a smaller proportion of GC B cells (figure 3A). Similarly, no differences were detected in the plasma cell frequency on day 14, but *Tnfsf4^−/−^ and Tnfsf4^fl/fl^(CD19)^−/−^* mice showed a significantly lower percentage of plasma cells on day 42 (figure 3B).

### B cell OX40L is essential for T_FH_ maturation

Having demonstrated the importance of OX40L in T cell activation and in plasma cell development, we investigated its possible role in T follicular helper cell (T_FH_) maturation. We identified the GC T_FH_ population as a subset of CD4+ T cells expressing CXCR5 and high levels of PD-1 (CXCR5+PD-1^hi^) (figure 3C,F), and in figure 3G the frequencies of splenic GC T_FH_ cells (as a proportion of CD4+ T cells) following immunisation are illustrated. There were fewer GC T_FH_ cells in the spleens of *Tnfsf4^−/−^ and Tnfsf4^fl/fl^(CD19)^−/−^* compared with wild-type mice during both the primary and secondary responses. In contrast, no differences were observed between controls and *Tnfsf4^fl/fl^(CD4)^−/−^* mice. Both *Tnfsf4^−/−^ and Tnfsf4^fl/fl^(CD19)^−/−^* mice showed a reduction in the expression levels of PD1 at both time points, and importantly displayed a greater frequency of CXCR5+ PD1^low^ cells (T_FH_ precursors) in the CD4+ population compared with control mice on day 42 (figure 3D,F). Interestingly, *Tnfsf4^−/−^* and *Tnfsf4^fl/fl^(CD19)^−/−^* mice also revealed a reduced number of CXCR5+ cells during the primary (day 14) but not the secondary response (day 42) (figure 3E).

### Lack of OX40L reduces T_FH_ number and ameliorates the lupus phenotype

In view of the genetic association of *TNFSF4* and SLE and the functional results outlined above, we investigated the effect of loss of OX40L in SLE using two different mouse models: a congenic model and a graft-versus-host model.

**Figure 4:**
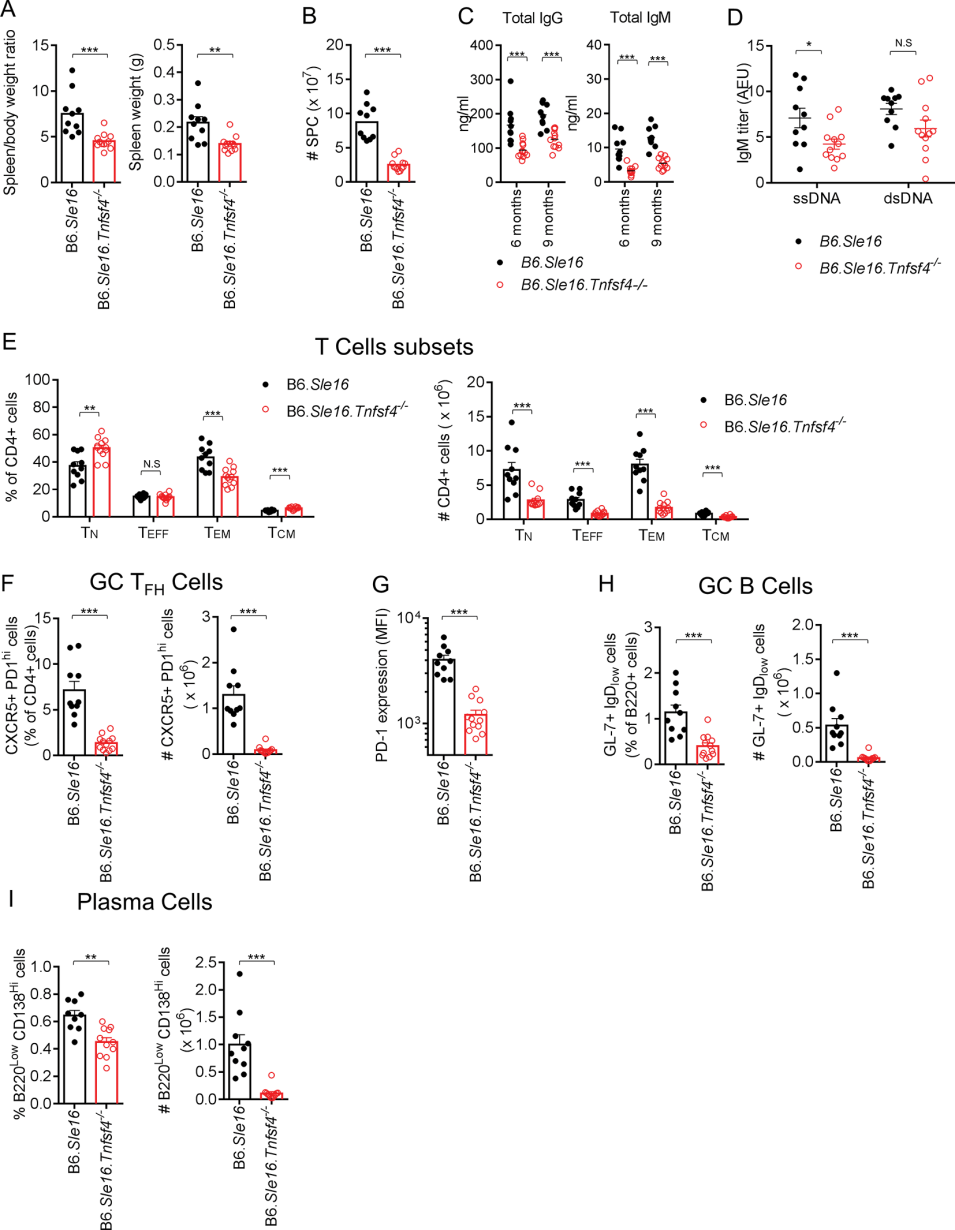
OX40L deficiency ameliorates the phenotype of B6.*Sle16* lupus-prone mice. Comparison between female B6.*Sle16* and *B6.Sle16.Tnfsf4^−/−^* female mice at 9 months of age. (A) Quantitation of spleen/body weight ratio and spleen weight. (B) Absolute number of cells per spleen. (C) Serum level of IgG and IgM at 6 and 9 months. (D) Titre of IgM anti-dsDNA and anti-ssDNA. (E) Quantitation of naïve (T_N_) (CD4+, CD62L+, CD44^low^), (T_EFF_) effector (CD4+, CD62L^low^, CD44^low^), T_EM_ effector/memory (CD4+, CD62L^low/neg^, CD44^hi^) and T_CM_ central/memory (CD4+, CD62L+, CD44^hi^) T cells expressed as a percentage of CD4+ cells and absolute number. (F) GC T_FH_ cells (CD4+, CXCR5+, PD-1^hi^) presented as frequency among the CD4+ population and absolute number. (G) PD-1 expression level in CD4+ cells assessed by FACS. (H) GC B cell (B220+, GL7+, IgD−) presented as frequency among the B220+ population and absolute number. (I) Percentage and absolute number of plasma cells (B220^low^, CD138^hi^). Each symbol represents an individual mouse. Bars indicate the mean±SEM N.S., not significant; *p<0.05, **p<0.01 and ***p<0.001 (t-test). dsDNA, double-stranded deoxyribonucleic acid; FACS, fluorescence-activated cell sorting; GC, germinal centre; ssDNA, single-stranded deoxyribonucleic acid.

*Tnfsf4^−/−^* mice were crossed with B6*.Sle16* lupus-prone mice, which are characterised by development of humoral autoimmunity associated with splenomegaly, high level of total IgG and IgM, autoantibodies production and glomerulonephritis linked to Ig and C3 deposition in the kidney.^31 32^ The resultant B6*.Sle16.Tnfsf4^−/−^* female animals were monitored for 9 months (figure 4). The absence of OX40L was associated with a marked reduction in splenomegaly (figure 4A,B) and a lower serum level of total IgG and IgM (figure 4C). No detectable levels of IgG anti-DNA were observed either in the knockout or the B6*.Sle16* control group. However, when we analysed IgM anti-ssDNA autoantibodies, a significant lower titre was observed in mice lacking OX40L compared with the B6*.Sle16* group (figure 4D). To investigate the effect of loss of OX40L on target organs, we quantified glomerular IgG, IgM and complement C3. As expected fluorescent quantification revealed significantly lower amount of IgG and IgM deposition in the glomerular in the absence of OX40L; in contrast a similar level of C3 deposition was observed (see online supplementary figure S3). Mice lacking OX40L had less T cell activation and higher proportions of central memory and naïve T cells (CD62L+ CD44^hi^ cells) (figure 4E). Consistent with the immune response data (figure 3), the *B6.Sle16.Tnfsf4^−/−^* showed a fivefold reduction, relative to the *B6.Sle16* mice, in the proportion of CD4+ T_FH_ cells (figure 4F), along with a dramatic reduction of PD-1 expression on CD4+ cells (figure 4G). Furthermore, the percentage of plasma cells and GC B cells (B220+ IgD GL7+) was also significantly lower in the absence of OX40L (figure 4H,I).

**Figure 5:**
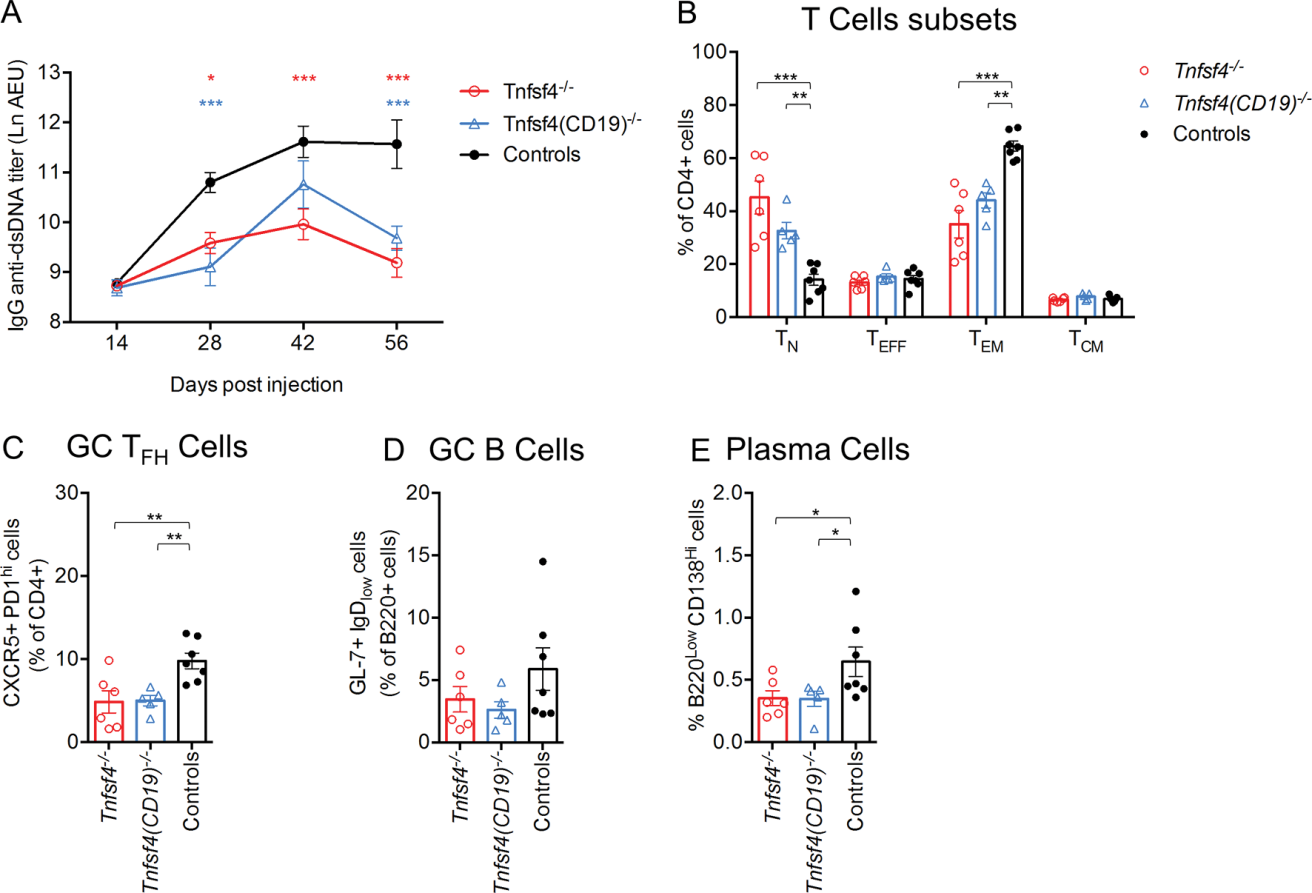
OX40L deficiency diminishes anti-dsDNA antibody production in the cGvHD model. Female wild-type controls, *Tnfsf4*^−/−^ and *Tnfsf4*(CD19)^−/−^ mice were injected intraperitoneally with 5×10^7^ splenocytes from B6.*H2^bm12^* female mice. Sera were collected on days 14, 28, 42 and 56. On day 56, spleens were collected and analysed by FACS. (A) Titre of IgG anti-dsDNA in the sera of injected mice at different time points. (B) Quantification of naïve (T_N_) (CD4+, CD62L+, CD44^low^), (T_EFF_) effector (CD4+, CD62L^low^, CD44^low^), (T_EM_) effector/memory (CD4+, CD62L^low/neg^, CD44^hi^) and (T_CM_) central/memory (CD4+, CD62L+, CD44^hi^) T cells expressed as percentage of CD4+ cells. (C) Quantification of GC T_FH_ cells (CD4+, CXCR5+, PD-1^hi^) presented as frequency among the CD4+ population. (D) Frequency of GC B cells (B220+,GL7+, IgD−) presented as frequency among the B220+ population. (E) Percentage of plasma cells (B220^low^, CD138^hi^). Each symbol represents data from an individual mouse. Bars indicate the mean±SEM. N.S., not significant; *p<0.05, **p<0.01 and ***p<0.001 (one-way analysis of variance). cGvHD, chronic graft-versus-host-disease; dsDNA, double-stranded deoxyribonucleic acid; FACS, fluorescence-activated cell sorting; GC, germinal centre.

We then used the I-A^bm12^ chronic graft-versus-host-disease (cGvHD) mouse model, in which an allogeneic interaction of T and B cells expressing different major histocompatibility complex (MHC) class II (I-A) induces an SLE-like phenotype.^37 38^ As shown in figure 5A, *Tnfsf4^−/−^* and *Tnfsf4^fl/fl^(CD19)^−/−^* mice injected with B6.*H2-Ab1^bm12^* splenocytes developed a lower titre of IgG anti-dsDNA compared with controls. In addition, both knockout groups showed a lower percentage of effector/memory T and T_FH_ cells (figure 5B,C). A trend towards a lower percentage of plasma and GC B cells was observed in both OX40L-deficient groups (figure 5D,E). Of note, no differences were seen between *Tnfsf4^−/−^* and *Tnfsf4^fl/fl^(CD19)^−/−^* mice, indicating that the observed differences are primarily due to the lack of OX40L on B cells.

## Discussion

The *TNFSF4* locus (that encodes OX40L) shows association with several autoimmune diseases; it has one of the most consistent and strongest genetic risk factors in SLE. OX40L has a well-established role in the activation and maintenance of T cell-mediated immune responses. However, the diversity of cells that express OX40L is such that a pathogenic mechanism relating the genetic findings to disease has not been clearly established. In this study, we generated B and CD4+ T cell OX40L conditional knockout mice, alongside a complete OX40L knockout, to explore and compare the function of OX40L on these cells.

Although a role for OX40L in the T-dependent antibody response has been suggested, conflicting results using different OX40L-deficient mice have been reported.^24 35^ These contradictory results may be partly explained by variability in genetic background.^39^ Our conditional knockout mice were on a pure C57BL/6 background and, in accord with the one study,^24^ our *Tnfsf4^−/−^* mice showed a reduced primary and secondary antibody response. However, while the *Tnfsf4^fl/fl^(CD19)^−/−^* mice showed the same phenotype as the *Tnfsf4^−/−^* mice, the *Tnfsf4^fl/fl^(CD4)^−/−^* mice had a normal secondary response, indicating that only OX40L expression by B cells is essential for the generation of an effective secondary humoral response and by implication B cell memory. We then investigated whether this defective humoral response was due to impaired T cell activation; as expected, *Tnfsf4^−/−^* mice showed lower percentage of T effector and T effector memory cells (figure 2). The same defect, although at a lower extent, was also shown by both conditional knockouts, despite the normal secondary response in *Tnfsf4^fl/fl^(CD4)^−/−^* mice. These results suggest that B cell OX40L may be involved in biological processes that promote memory responses that are independent of T cell activation.

T cell-dependent B cell immune response involves both an extrafollicular response, which generates short-lived plasma cells and an early wave of low-affinity antibody production, and a GC response, which gives rise to long-lived plasma or memory cells and a later wave of high-affinity antibodies. OX40L has been previously suggested to be essential for the development of high-affinity Ig-producing plasma cells^26^; however, no further evidence has been subsequently reported. In our study, alongside an impaired memory response (figure 1C), there were fewer plasma cells on day 42 in *Tnfsf4^−/−^* and *Tnfsf4^fl/fl^(CD19)^−/−^* mice (figure 3B), which suggests that B cell OX40L contributes to an effective GC reaction.

We show that *Tnfsf4^−/−^* and *Tnfsf4^fl/fl^(CD19)^−/−^* mice have a lower percentage of GC T_FH_, one of the main contributors to the GC reaction. The development of mature GC T_FH_, which characteristically expresses CXCR5, along with high levels of the surface receptors ICOS, CD40 ligand (CD40L), PD-1 and importantly OX40,^40 41^ includes two stages: after activation, a fraction of CD4+ T cells migrate towards B cell follicles by upregulating the chemokine receptor CXCR5, and these T_FH_ precursors then interact with antigen-presenting B cells at the border of the B cell follicle and T cell zone and fully maturate into functional GC T_FH_ cells.^41^ In particular OX40L has been shown to be essential for the expression of CXCR5 and the consequent migration of T cells at the T/B border of B cell follicles,^22 42 43^ providing the first evidence of the role of OX40L in this process. Our results corroborate this finding; we found that fewer CXCR5+ T cells were generated during the primary response in *Tnfsf4^−/−^* and *Tnfsf4^fl/fl^(CD19)^−/−^* mice (figure 3E). Whether OX40L-OX40 signal is responsible for the induction, maturation or maintenance of T_FH_ cells and which cell types expressing OX40L are necessary is still unclear; however, a recent work by Tahiliani and colleagues^44^ shows a markedly diminished humoral response and production of fewer T_FH_ cells in OX40 KO mice following immunisation with vaccinia virus. In particular the authors show a direct association between OX40+ T_FH_ cells and OX40L-expressing DCs and B cells at the T/B borders and GC providing supportive evidence to how a sustained OX40L-OX40 signal on T_FH_ cells is necessary for the induction of T_FH_ cells and their maturation to maintain a proper GC reaction. In our study, the reduced numbers of T_FH_ cells in *Tnfsf4^−/−^* and *Tnfsf4^fl/fl^(CD19)^−/−^* mice were accompanied by an increase in CXCR5+ PD1^low^ cells during the secondary response (figure 3F,G). Since low levels of cell-surface PD1 have been shown to characterise T_FH_ precursor cells,^45^ our data suggest a novel role for OX40L on B cells: after activation by DCs, immature T_FH_ cells migrate towards the T/B borders of the B cells follicles, where activated antigen presenting B cells induced their maturation into the GC T_FH_ resident state and their maintenance by sustaining OX40L-OX40 signalling.

*TNFSF4* has been reproducibly associated with SLE.^4 5^ A recent important study from Jacquemin and colleagues^28^ demonstrated that stimulation through OX40 induced T cells to express T_FH_ cells-specific genes such as Bcl6 and CXCR5. They also observed a positive correlation between disease activity, percentage of blood T_FH_ cells and frequency of OX40L+myeloid APC, suggesting OX40L-OX40 axis as a contributor factor in the aberrant T_FH_ response observed in SLE.^46 47^ However, the ability to study tissue T_FH_ in humans is limited. In our study, although in a murine model, the generation of T_FH_ cells in the spleen is similarly impeded in the B cell conditional knockout and in the germline *Tnfsf4* knockout, indicating the importance of B cell OX40L. In the human study,^28^ there was no correlation between blood B cells expressing OX40L and T_FH_ cells. However, this lack of correlation could be a consequence of the compartmentalisation of activated B cells expressing OX40L in the secondary lymphoid organs rather than an evidence of their lack of involvement in the development of pathogenic T_FH_ cells in SLE.

In our study, to elucidate the role of OX40L in SLE, we used two different SLE mouse models, and in particular the GvHD model was chosen to investigate the role of OX40L on B cells during the B–T cell interaction. In both models of systemic autoimmunity, the lack of OX40L-OX40 signalling was associated with amelioration of the disease phenotype, as shown by a reduced production of anti-dsDNA autoantibodies and Ig kidney deposition together with reduced numbers of GC T_FH_ (figures 4F and 5C) and plasma cells (figures 4I and 5E). These data suggest that OX40L supports the expression of the disease phenotype as well as autoantibody production. This conclusion is further strengthened by the observation that blockade of OX40L reduces degree of proteinuria associated with glomerulonephritis in an accelerated murine model.^48^

The results presented in this paper support a mechanism by which genetically determined elevated expression of OX40L predisposes to SLE via increased B cell expression, which in turn supports T_FH_ development. In light of the argument that genetic factors augment the likelihood of success with a drug target,^49^ our data strongly support exploration of this therapeutic strategy. It is potentially important for optimal treatment to know which OX40L-expressing cell types should be targeted, and the defined risk alleles at *TNFSF4* further raise the possibility that genetic screening may identify individuals most likely to benefit from OX40L inhibition.

## References

[1] Mohan C, Putterman C Genetics and pathogenesis of systemic lupus erythematosus and lupus nephritis. Nat Rev Nephrol 2015;11:329–41. 10.1038/nrneph.2015.33 25825084

[2] Tsokos GC Systemic lupus erythematosus. N Engl J Med 2011;365:2110–21. 10.1056/NEJMra1100359 22129255

[3] Harley JB, Alarcón-Riquelme ME, Criswell LA, et al Genome-wide association scan in women with systemic lupus erythematosus identifies susceptibility variants in ITGAM, PXK, KIAA1542 and other loci. Nat Genet 2008;40:204–10. 10.1038/ng.81 18204446 PMC3712260

[4] Bentham J, Morris DL, Graham DSC, et al Genetic association analyses implicate aberrant regulation of innate and adaptive immunity genes in the pathogenesis of systemic lupus erythematosus. Nat Genet 2015;47:1457–64. 10.1038/ng.3434 26502338 PMC4668589

[5] Cunninghame Graham DS, Graham RR, Manku H, et al Polymorphism at the TNF superfamily gene TNFSF4 confers susceptibility to systemic lupus erythematosus. Nat Genet 2008;40:83–9. 10.1038/ng.2007.47 18059267 PMC3705866

[6] Nordmark G, Kristjansdottir G, Theander E, et al Association of EBF1, FAM167A(C8orf13)-BLK and TNFSF4 gene variants with primary Sjögren’s syndrome. Genes Immun 2011;12:100–9. 10.1038/gene.2010.44 20861858

[7] Faraco J, Lin L, Kornum BR, et al ImmunoChip study implicates antigen presentation to T cells in narcolepsy. PLoS Genet 2013;9:e1003270 10.1371/journal.pgen.1003270 23459209 PMC3573113

[8] Gourh P, Arnett FC, Tan FK, et al Association of TNFSF4 (OX40L) polymorphisms with susceptibility to systemic sclerosis. Ann Rheum Dis 2010;69:550–5. 10.1136/ard.2009.116434 19778912 PMC2927683

[9] Bossini-Castillo L, Broen JC, Simeon CP, et al A replication study confirms the association of TNFSF4 (OX40L) polymorphisms with systemic sclerosis in a large European cohort. Ann Rheum Dis 2011;70:638–41. 10.1136/ard.2010.141838 21187296

[10] Manku H, Langefeld CD, Guerra SG, et al Trans-ancestral studies fine map the SLE-susceptibility locus TNFSF4. PLoS Genet 2013;9:e1003554 10.1371/journal.pgen.1003554 23874208 PMC3715547

[11] Grundberg E, Small KS, Hedman ÅK, et al Multiple Tissue Human Expression Resource (MuTHER) Consortium. Mapping cis- and trans-regulatory effects across multiple tissues in twins. Nat Genet 2012;44:1084–9. 10.1038/ng.2394 22941192 PMC3784328

[12] Linton PJ, Bautista B, Biederman E, et al Costimulation via OX40L expressed by B cells is sufficient to determine the extent of primary CD4 cell expansion and Th2 cytokine secretion in vivo. J Exp Med 2003;197:875–83 http://www.jem.org/cgi/doi/ 10.1084/jem.20021290 12668647 PMC2193894

[13] Karulf M, Kelly A, Weinberg AD, et al OX40 ligand regulates inflammation and mortality in the innate immune response to sepsis. J Immunol 2010;185:4856–62. 10.4049/jimmunol.1000404 20844189 PMC3622718

[14] Jenkins SJ, Perona-Wright G, Worsley AG, et al Dendritic cell expression of OX40 ligand acts as a costimulatory, not polarizing, signal for optimal Th2 priming and memory induction in vivo. J Immunol 2007;179:3515–23. 10.4049/jimmunol.179.6.3515 17785785

[15] Soroosh P, Ine S, Sugamura K, et al OX40-OX40 ligand interaction through T cell-T cell contact contributes to CD4 T cell longevity. J Immunol 2006;176:5975–87. 10.4049/jimmunol.176.10.5975 16670306

[16] Mendel I, Shevach EM Activated T cells express the OX40 ligand: requirements for induction and costimulatory function. Immunology 2006;117:196–204. 10.1111/j.1365-2567.2005.02279.x 16423055 PMC1782207

[17] Webb GJ, Hirschfield GM, Lane PJ OX40, OX40L and Autoimmunity: a Comprehensive Review. Clin Rev Allergy Immunol 2016;50:312–32. 10.1007/s12016-015-8498-3 26215166

[18] Croft M, So T, Duan W, et al The significance of OX40 and OX40L to T-cell biology and immune disease. Immunol Rev 2009;229:173–91. 10.1111/j.1600-065X.2009.00766.x 19426222 PMC2729757

[19] Melero I, Hirschhorn-Cymerman D, Morales-Kastresana A, et al Agonist antibodies to TNFR molecules that costimulate T and NK cells. Clin Cancer Res 2013;19:1044–53. 10.1158/1078-0432.CCR-12-2065 23460535 PMC4397897

[20] Zaini J, Andarini S, Tahara M, et al OX40 ligand expressed by DCs costimulates NKT and CD4+ Th cell antitumor immunity in mice. J Clin Invest 2007;117:3330–8. 10.1172/JCI32693 17975668 PMC2045612

[21] Baumann R, Yousefi S, Simon D, et al Functional expression of CD134 by neutrophils. Eur J Immunol 2004;34:2268–75. 10.1002/eji.200424863 15259024

[22] Brocker T, Gulbranson-Judge A, Flynn S, et al CD4 T cell traffic control: in vivo evidence that ligation of OX40 on CD4 T cells by OX40-ligand expressed on dendritic cells leads to the accumulation of CD4 T cells in B follicles. Eur J Immunol 1999;29:1610–6. 10.1002/(SICI)1521-4141(199905)29:05<1610::AID-IMMU1610>3.0.CO;2-8 10359115

[23] Murata K, Nose M, Ndhlovu LC, et al Constitutive OX40/OX40 ligand interaction induces autoimmune-like diseases. J Immunol 2002;169:4628–36. 10.4049/jimmunol.169.8.4628 12370402

[24] Murata K, Ishii N, Takano H, et al Impairment of antigen-presenting cell function in mice lacking expression of OX40 ligand. J Exp Med 2000;191:365–74. 10.1084/jem.191.2.365 10637280 PMC2195745

[25] Ohshima Y, Tanaka Y, Tozawa H, et al Expression and function of OX40 ligand on human dendritic cells. J Immunol 1997;159:3838–48. 9378971

[26] Stüber E, Strober W The T cell-B cell interaction via OX40-OX40L is necessary for the T cell-dependent humoral immune response. J Exp Med 1996;183:979–89. 10.1084/jem.183.3.979 8642301 PMC2192367

[27] Stüber E, Neurath M, Calderhead D, et al Cross-linking of OX40 ligand, a member of the TNF/NGF cytokine family, induces proliferation and differentiation in murine splenic B cells. Immunity 1995;2:507–21. 10.1016/1074-7613(95)90031-4 7749983

[28] Jacquemin C, Schmitt N, Contin-Bordes C, et al OX40 Ligand Contributes to Human Lupus Pathogenesis by Promoting T Follicular Helper Response. Immunity 2015;42:1159–1170. 10.1016/j.immuni.2015.05.012 26070486 PMC4570857

[29] Liu P, Jenkins NA, Copeland NG, et al A Highly Efficient Recombineering-Based Method for Generating Conditional Knockout Mutations A Highly Efficient Recombineering-Based Method for Generating Conditional Knockout Mutations. Genome Res 2003;84:476. 10.1101/gr.749203 PMC430283 12618378

[30] Han S, Yang K, Ozen Z, et al Enhanced differentiation of splenic plasma cells but diminished long-lived high-affinity bone marrow plasma cells in aged mice. J Immunol 2003;170:1267–73. 10.4049/jimmunol.170.3.1267 12538685

[31] Carlucci F, Cortes-Hernandez J, Fossati-Jimack L, et al Genetic dissection of spontaneous autoimmunity driven by 129-derived chromosome 1 Loci when expressed on C57BL/6 mice. J Immunol 2007;178:2352–60. 10.4049/jimmunol.178.4.2352 17277141

[32] Bygrave AE, Rose KL, Cortes-Hernandez J, et al Spontaneous autoimmunity in 129 and C57BL/6 mice-implications for autoimmunity described in gene-targeted mice. PLoS Biol 2004;2:e243 10.1371/journal.pbio.0020243 15314659 PMC509305

[33] Wolfer A, Bakker T, Wilson A, et al Inactivation of Notch 1 in immature thymocytes does not perturb CD4 or CD8T cell development. Nat Immunol 2001;2:235–41. 10.1038/85294 11224523

[34] Rickert RC, Roes J, B lymphocyte-specific RK Cre-mediated mutagenesis in mice. Nucleic Acids Res 1997;25:1317–8. 9092650 10.1093/nar/25.6.1317 PMC146582

[35] Chen AI, McAdam AJ, Buhlmann JE, et al Ox40-ligand has a critical costimulatory role in dendritic cell:T cell interactions. Immunity 1999;11:689–98. 10.1016/S1074-7613(00)80143-0 10626891

[36] Soroosh P, Ine S, Sugamura K, et al Differential requirements for OX40 signals on generation of effector and central memory CD4+ T cells. J Immunol 2007;179:5014–23. 10.4049/jimmunol.179.8.5014 17911586

[37] Morris SC, Cheek RL, Cohen PL, et al Autoantibodies in chronic graft versus host result from cognate T-B interactions. J Exp Med 1990;171:503–17. 10.1084/jem.171.2.503 2303783 PMC2187721

[38] Eisenberg RA, Via CS T cells, murine chronic graft-versus-host disease and autoimmunity. J Autoimmun 2012;39:240–7. 10.1016/j.jaut.2012.05.017 22704961 PMC3578438

[39] Rabieyousefi M, Soroosh P, Satoh K, et al Indispensable roles of OX40L-derived signal and epistatic genetic effect in immune-mediated pathogenesis of spontaneous pulmonary hypertension. BMC Immunol 2011;12:67 10.1186/1471-2172-12-67 22171643 PMC3269997

[40] Ma CS, Deenick EK, Batten M, et al The origins, function, and regulation of T follicular helper cells. J Exp Med 2012;209:1241–53. 10.1084/jem.20120994 22753927 PMC3405510

[41] Ueno H, Banchereau J, Vinuesa CG Pathophysiology of T follicular helper cells in humans and mice. Nat Immunol 2015;16:142–52. 10.1038/ni.3054 25594465 PMC4459756

[42] Walker LS, Gulbranson-Judge A, Flynn S, et al Compromised OX40 function in CD28-deficient mice is linked with failure to develop CXC chemokine receptor 5-positive CD4 cells and germinal centers. J Exp Med 1999;190:1115–22. 10.1084/jem.190.8.1115 10523609 PMC2195670

[43] Flynn S, Toellner KM, Raykundalia C, et al CD4 T cell cytokine differentiation: the B cell activation molecule, OX40 ligand, instructs CD4 T cells to express interleukin 4 and upregulates expression of the chemokine receptor, Blr-1. J Exp Med 1998;188:297–304. 10.1084/jem.188.2.297 9670042 PMC2212448

[44] Tahiliani V, Hutchinson TE, Abboud G, et al OX40 Cooperates with ICOS To Amplify Follicular Th Cell Development and Germinal Center Reactions during Infection. J Immunol 2017;198:218–28. 10.4049/jimmunol.1601356 27895177 PMC5173420

[45] Lee SK, Rigby RJ, Zotos D, et al B cell priming for extrafollicular antibody responses requires Bcl-6 expression by T cells. J Exp Med 2011;208:1377–88. 10.1084/jem.20102065 21708925 PMC3135363

[46] Ueno H Human Circulating T Follicular Helper Cell Subsets in Health and Disease. J Clin Immunol 2016;36(Supp 1):34–9. 10.1007/s10875-016-0268-3 26984851

[47] Crotty S T follicular helper cell differentiation, function, and roles in disease. Immunity 2014;41:529–42. 10.1016/j.immuni.2014.10.004 25367570 PMC4223692

[48] Sitrin J, Suto E, Wuster A, et al The Ox40/Ox40 Ligand Pathway Promotes Pathogenic Th Cell Responses, Plasmablast Accumulation, and Lupus Nephritis in NZB/W F1 Mice. J Immunol 2017:ji1700608 10.4049/jimmunol.1700608 PMC5544932 28696253

[49] Plenge RM, Scolnick EM, Altshuler D Validating therapeutic targets through human genetics. Nat Rev Drug Discov 2013;12:581–94. 10.1038/nrd4051 23868113

