# Supplementary material for: B cell OX40L supports T follicular helper cell development and contributes to SLE pathogenesis
Source: Ann Rheum Dis. 2017 Aug 17;76(12):2095–103. doi: 10.1136/annrheumdis-2017-211499 (PMC5705841; doi:10.1136/annrheumdis-2017-211499)
Supplement: Supplementary file 1 [file annrheumdis-2017-211499supp001.pdf]

Supplementary figure S1

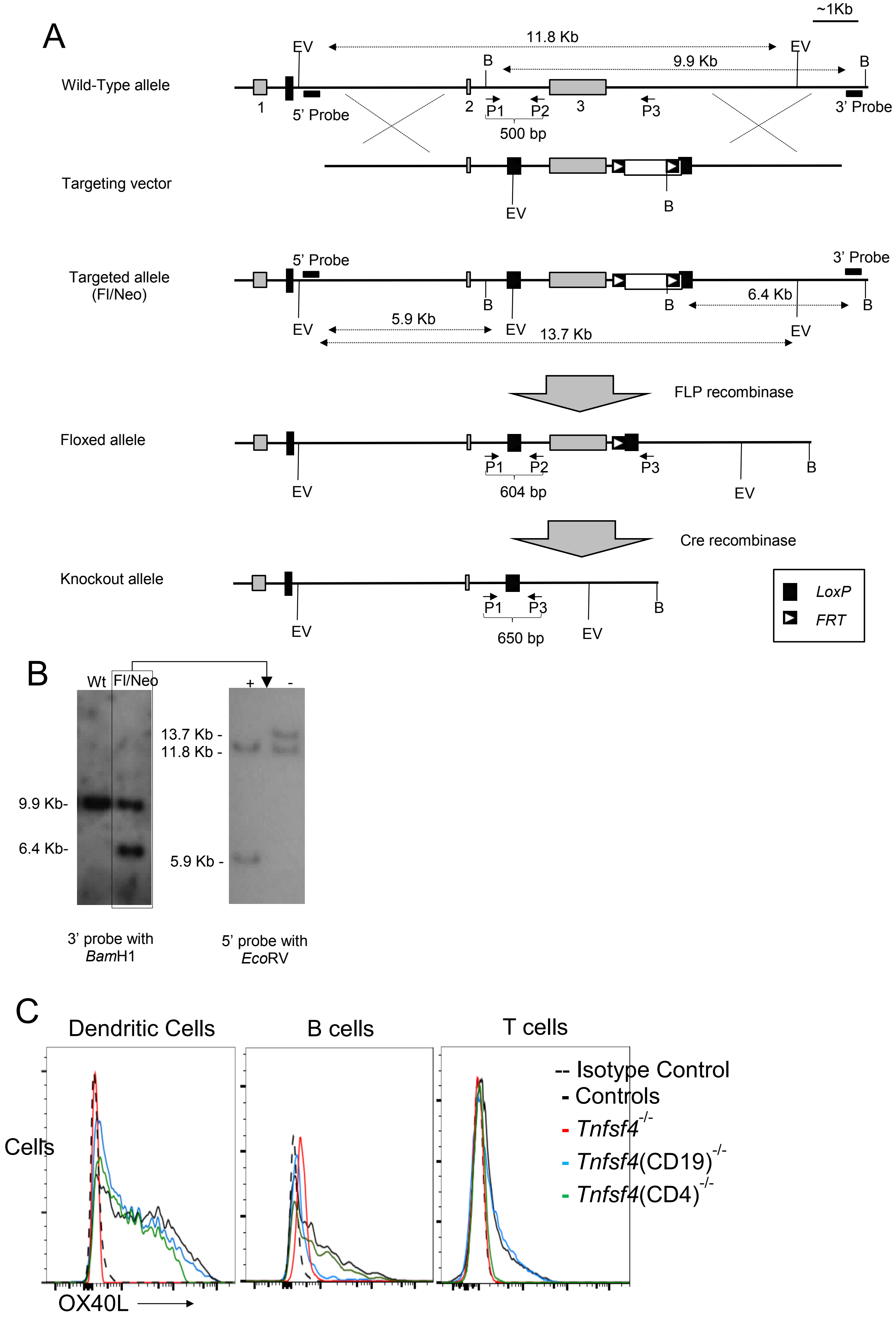

Supplementary Figure S1 Generation and validation of *Tnfsf4* conditional knockout mouse

(A) Schematic representation of the strategy used to generate a conditional *Tnfsf4* mouse. The targeting vector was generated by inserting two loxP sites (black arrows) on either site of exon 3 of the *Tnfsf4* gene. The target allele contained a neomycin–resistant (Neo) positive selectable marker flanked by flippase recombinase recognition target sites (white arrows). Cre recombinase-mediated recombination between the loxP sites results in excision of exon 3, which contains the majority of the gene coding sequence, leading to a null allele. P1, P2 and P3 indicate the location of the primers used for genotyping screening; *Bam*HI (B) and *Eco*RV (EV) are the restriction enzymes used for Southern blot analysis **(B)** of positive ES cells clone with an external 3' and 5' probe. **(C)** FACS analysis of stimulated dendritic- and B- cells for expression of OX40L in wild-type controls (black line), *Tnfsf4*$^{-/-}$ (red line), *Tnfsf4*$^{fl/fl}$/CD19-cre (blue line) and *Tnfsf4*$^{fl/fl}$/CD4-cre (green line) mice.

Supplementary figure S2

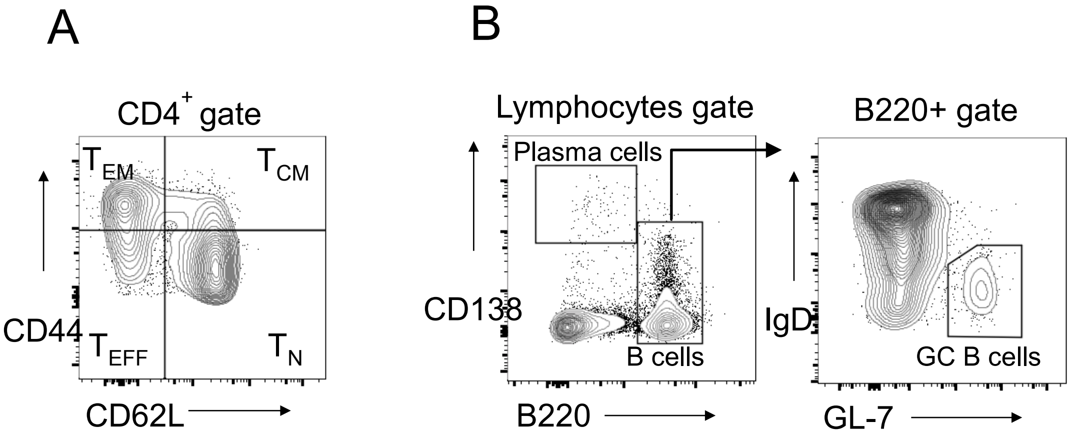

Supplementary Figure S2 Gating strategy for the analysis of T- and B- cells subsets

(A) Gating strategy for the analysis of T cells subsets. **(B)** Gating strategy for the analysis of B cells subsets.

Supplementary figure S3

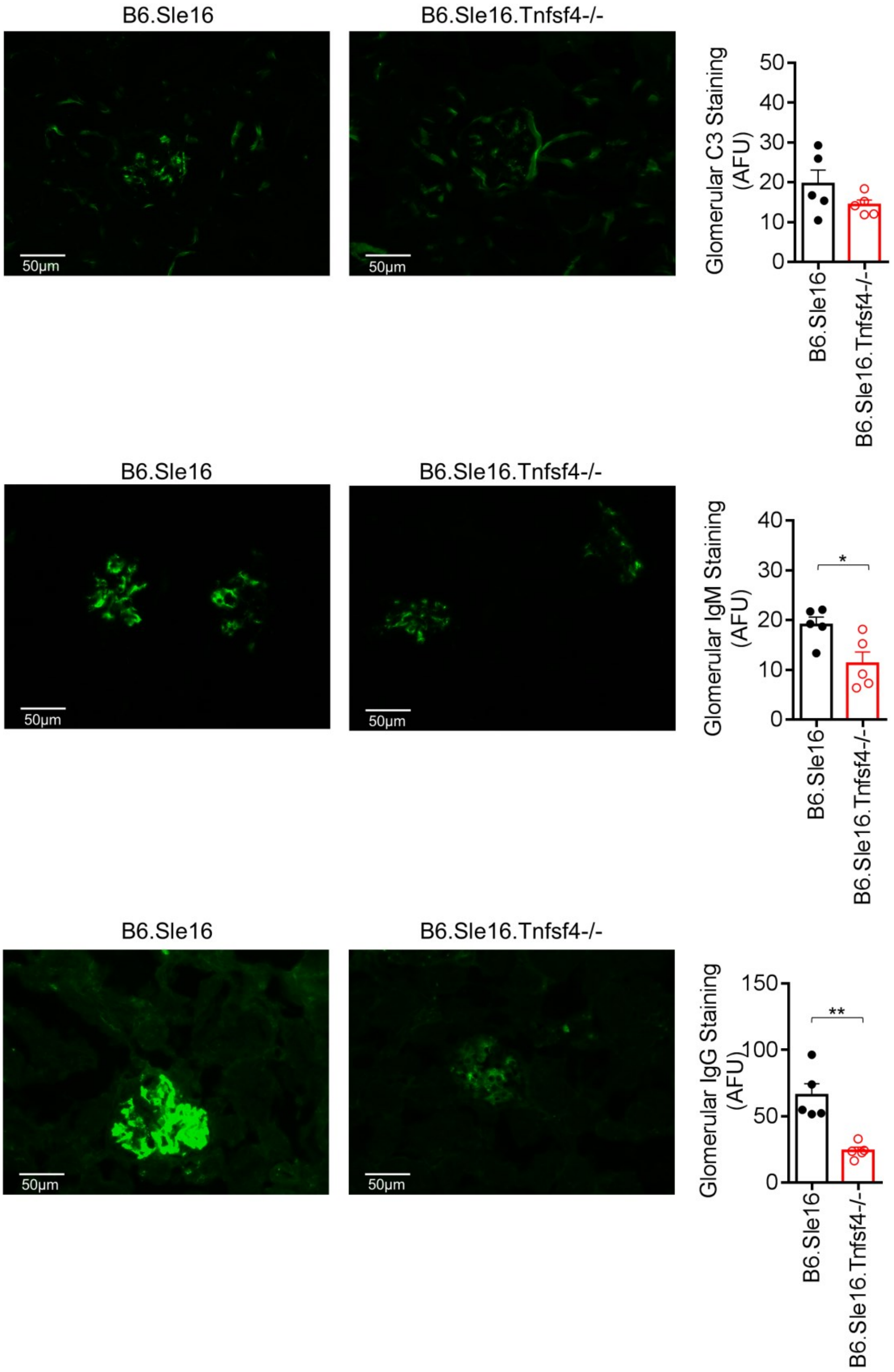

Supplementary Figure S3 OX40L deficiency ameliorates the phenotype of B6.Sle16 lupus-prone mice

Representative glomerular staining of mouse C3 (Top), IgM (middle) and IgG (Bottom) deposition detected by immunofluorescence in kidney sections of *B6.Sle16* (left column) and *B6.Sle16.Tnfsf4*$^{-/-}$(middle column); magnification 40X, scale bars are provided. Quantitative analysis (left column) of immunofluorescent sections revealed lower deposition of IgM and IgG in *B6.Sle16.Tnfsf4*$^{-/-}$ compered to B6.Sle16 mice. Each symbol represents an individual mouse. Bars indicate the mean ±SEM. N.S., not significant; *P < 0.05, **P < 0.01 and ***P < 0.001 (Kruskal-Wallis test).

Supplementary Table 1

Mice	Primer ID	Primer sequence	PCR band size
Tnfsf4 mice	P1	ATAAGCGGCCGCAGCACATGGCTATATCTTTGA	Wild Type: 500 bp<br>Floxed: 604 bp<br>Knock-out allele: 650bp
	P2	CTCGGTACCCAGCTGTGTCTGTGGGGTAGA	
	P3	CTCGGTACCGAGCACTTGCATAGTAACTGG	
CD19-Cre	CRE1	CGAGTGATGAGGTTCGCAAG	Wild Type: 170 bp<br>Mutation: 390bp
	CRE2	TGAGTGAACGAACCTGGTCG	
CD4-Cre	CRE Fwd	CGATGCAACGAGTGATGAGG	Transgenic mice: 400bp
	CRE Rev	GCATTGCTGTCACTTGGTCGT	
